# The risk assessment of relapse among newly enrolled participants in methadone maintenance treatment: A group-LASSO based Bayesian network study

**DOI:** 10.3389/fpubh.2022.1032217

**Published:** 2023-01-17

**Authors:** Xijia Tang, Chaonan Fan, Chijie Wang, Wenjuan Wang, Zouxiang Chen, Chaofan Xu, Li Ling

**Affiliations:** ^1^Department of Medical Statistics, School of Public Health, Sun Yat-sen University, Guangzhou, Guangdong, China; ^2^Clinical Research Design Division, Clinical Research Center, Sun-Yat sen Memorial Hospital, Sun Yat-sen University, Guangdong, China

**Keywords:** methadone maintenance treatment, relapse, treatment duration, risk assessment, Bayesian networks

## Abstract

**Background:**

Relapse is a great barrier to improving the effectiveness of methadone maintenance treatment (MMT). Participants with different treatment durations could vary in their compliance with MMT, which may lead to different levels of relapse risk. This study aims to identify the risk factors for relapse and assess the relapse risk of MMT participants of different treatment durations.

**Method:**

This retrospective study used data collected from seven MMT clinics in Guangdong Province, China, from January 2010 to April 2017. Newly enrolled participants who received 6 (*n* = 903) and 12 (*n* = 710) months of consecutive treatment with complete data were included. We selected significant risk factors for relapse through the group lasso regression and then incorporated them into Bayesian networks to reveal relationships between factors and predict the relapse risk.

**Results:**

The results showed that participants who received 6-month treatment had a lower relapse rate (32.0%) than those of 12-month treatment (39.0%, *P* < 0.05). Factors including personal living status and daily methadone dose were only influential to those who received the 6-month treatment. However, age, age at the initial drug use, HIV infection status, sexual behaviors, and continuous treatment days were common factors of both durations. The highest relapse risk for those after the 6-month treatment was inferred as 66.7% while that of the 12-month treatment was 83.3%. Farmers and those who have high accessibility to MMT services may require additional attention.

**Conclusion:**

It is necessary to implement targeted interventions and education based on the treatment durations of participants to decrease the relapse rate. Meanwhile, those about HIV/sexually transmitted infection prevention and anti-narcotics should be held in the whole process.

## Introduction

Methadone maintenance treatment (MMT) is one of the safest and most cost-effective substitution therapies to manage opioid dependence, which has been implemented in 84 countries and territories worldwide (1). It is thought to be effective in reducing high-risk behavior of opioid users, such as unprotected sex, syringe sharing, and contributing greatly to preventing human immunodeficiency virus (HIV) infection/acquired immunodeficiency syndrome (AIDS). It also assists in crime reduction and enhances social productivity among opioid users (2, 3). China has the largest MMT program in the world, covering 29 of 34 provinces nationwide by 2020 (4, 5), and it has served nearly 10% of MMT participants globally. However, for most countries implementing MMT (6, 7), including China, relapse has always been a common and great barrier to its development. It is a complex consequence led by multiple factors and will put participants at higher risk of HIV infection and overdose, and negatively affects their return to day-to-day life (8). It was reported that 20–57% of MMT participants would relapse in the first 6 months and the rate would increase when they were treated longer (9–11). A study conducted in Iran showed that the relapse rate was approximately 30–50% after 12-month MMT (12) and was as high as 94% when the duration was extended to 18 to 36 months (13). Similarly, 31% of Chinese MMT participants reported relapse in the first 6 months and the rate rose to 56% after 12-month consecutive treatment (5).

Therefore, participants receiving MMT over different durations may be at different risk levels of relapse. Identifying high-risk groups and assessing the risk based on their treatment durations could help healthcare providers to target more aggressive adjunct therapies and increase the effectiveness of MMT. Previous studies have explored much about risk factors of relapse (14–19) and developed several tools for risk assessment (5, 16); however, there are still research gaps to be filled.

On the one hand, there were some commonly recognized risk factors including the age of onset of opioid abuse, frequent injection, insufficient methadone dose, and poor social support (5, 16, 20). Specifically, the younger age at the onset of substance use may increase the relapse risk due to genetic and environmental influences (21). Those who reported frequent injection behavior may require higher levels of opioids and suffer more social marginalization, making them difficult to manage on the MMT (16). In addition, the insufficient dose could not effectively control the euphoric effects of heroin and then cause withdrawal symptoms, which drives the participants to relapse (22). However, whether these factors and their effect size will vary along with treatment durations remains unclear.

On the other hand, the developed tools were usually constructed using the logistic regression model (23) or the Cox proportion hazard model (24). These methods did not perform well in dealing with the dependence on variables, which is the character of relapse. To solve this problem, previous studies chose to remove the variables with dependence (25) and apply stepwise (26) or penalized regression (27). The least absolute shrinkage and selection operator (LASSO) is one of the penalized regressions, and the group lasso regression, as an extension, can select variables at the group level instead of a single dummy variable (28), which suits analyzing the complicated outcome such as relapse. In addition, traditional methods could not reveal the association between variables and quantify the risk at the individual level either. Bayesian networks (BNs) are graphical models that describe the probability relationship between a set of variables (29). BNs have been increasingly used to predict the risk of particular diseases, such as accurate kidney injury (28) and chronic obstructive pulmonary disease (30), while none was found in the field of MMT yet.

In this study, we combined these two methods to triage the high-risk groups and assess the relapse risk of different treatment durations. Hopefully, our findings will be instructive for developing individualized treatment plans for MMT participants to improve their compliance and decrease the relapse rate.

## Materials and methods

### Study design and data source

This is a retrospective study that used secondary data to assess the risk of relapse among newly enrolled MMT participants of different treatment durations. We selected Guangdong province as the study setting, as it had the highest rate of drug crimes (31) and 30% of registered drug users in China by 2018 (32), and it has established 66 MMT clinics, ranking 4th place in the country (4). We employed a two-stage stratified sampling methodology by first choosing eight cities in Guangdong with different levels of economic development, and then randomly selecting one or two MMT clinics from each city. Accordingly, 10 MMT clinics were selected and three of them were excluded for the lack of baseline information. A total of seven MMT clinics were finally included.

The data analyzed in this study were derived from the web-based National Unified MMT management system.

### Data collection

We collected the unidentifiable baseline information of newly enrolled participants from January 2010 to April 2017 from a questionnaire developed by the National Working Group on MMT. All participants gave their written consent for their information to be stored in the national web-based MMT system and allowed to use for research (33). This survey was completed by the clinic staff and included demographic information, sex, drug use-related behavior, and infection status (HIV and HCV). The HIV antibody was initially screened by the colloidal gold method, and positive samples were confirmed using Western blotting. The HCV antibody test was conducted using the enzyme-linked immunosorbent assay method. Both tests were conducted at enrollment. The daily methadone dose and the result of monthly urine morphine were also collected. All the aforementioned tests were conducted by doctors of the MMT clinic or the local center for disease control and prevention (CDC).

Participants were eligible if they were >18 years old, provided written informed consent, and were diagnosed as opioid-dependent using the Chinese Classification of Mental Diseases Criteria (third version). The description definition of these criteria was improved based on ICD-10 and the diagnostic criteria were referred to ICD-10 and *the Diagnostic and Statistical Manual of Mental Disorder*, 4th edition (DSM-IV) (34), but it had a unique definition of some disorders, such as culturally related diagnoses. Participants who were re-enrolled or referred from another MMT clinic, who were residents outside Guangdong, and who had an incomplete record of the daily dose and urine morphine test were excluded.

### Outcome and definitions of calculated variables

#### Relapse

Our primary outcome is whether the participants relapsed during the MMT. Relapse in this study was defined as showing at least two consecutive positive results of the monthly urine morphine test during treatment, in line with the relevant literature and guidelines (11, 20, 35–37). The first 10 to 30 days after the enrollment was usually considered the adjustment phase (38) and there was still a relatively high possibility for participants to use heroin during this period. Therefore, the result of the urine morphine test in the first month was excluded from the analysis, and the actual treatment durations were extended to 7 and 13 months accordingly.

#### Initial daily methadone dose

The initial daily methadone dose indicated the average daily methadone dose of the first week of MMT, rather than that of the first day, to provide a stable result.

#### Continuous treatment days

Drop-out was usually defined as being absent from MMT for more than 14 consecutive days (39). The continuous treatment days were days participants resumed MMT after their last drop-out (18). For those who did not drop out, continuous treatment days equaled their entire treatment duration. This is an indicator of the compliance of participants.

### Statistical analysis

To assess the risk of relapse among MMT participants, we used the group lasso regression to select significant risk factors and then incorporate them into Bayesian networks to make risk predictions.

To be more detailed, the lasso regression applies the L1 norm to the unknown coefficient vector and the coefficients with smaller absolute values would be directly compressed to 0, those with non-zero coefficients were considered significant variables (40). Factors associated with relapse are usually dependent on each other while the lasso regression will treat them as independent variables (41). We used the group lasso instead to select predefined grouping variables (28). The parameter estimation of the group lasso is presented as follows:


$$\begin{array}{l} {\hat{\beta }}^{GrLasso}=\arg_{\beta }\min\left\{{\overset{n}{\underset{i=1}{\sum }}\frac{1}{2}\left(y_{i}-\overset{p}{\underset{j=1}{\sum }}x_{ij}\beta_{j}\right)}^{2}\right\}+n\lambda \overset{p}{\underset{j=1}{\sum }}{||\beta }_{j}|| \end{array}$$

Where *j* presents the number of groups of the variables and each group has *p*_1_*, p*_2_…*p*_*j*_ levels. λ is the adjusted parameter, which was used to control the extent of the penalty, and the optimal parameter was selected by k-folded cross-validation. The minimum cross-validation error refers to the best model (28).

The selected variables were then used to establish Bayesian networks (BNs). BNs consist of a directed acyclic graph (DAG) and conditional probability tables (CPT). DAG is constructed based on the assumption of conditional independence, and the probability dependence among nodes is quantified by CPT, specified as follows:


$$\begin{array}{l} P\left(X\right)=\overset{N}{\underset{i=1}{\prod }}P\left(X_{i}{|\prod }_{X_{i}};\Theta_{X_{i}}\right) \end{array}$$

Where *P(X)* indicates the probability of the outcome, Θ*x*_*i*_ represents the parameter of node *X*_*i*_, and ∏_*X*_*i*__ means the parent node set of *X*_*i*_. A complete BN model is established through parameter learning and structure learning. In this study, we chose maximum likelihood estimation as the parameter learning method. Tabu-search was chosen as the structure learning method as it can avoid the locally optimal solution (29).

The Pearson chi-square test or Fisher's exact test (when the expected frequencies were <1 in either cell or <5 of over 20% of cells) was used to compare the difference in the distribution of categorical variables between participants who relapsed and those who did not, and α was set as 0.1. The area under the receiver operating characteristic curve (AUC) was applied to estimate the prediction capacity of the models. Missing values were imputed using multiple imputations, as it suited most types of data (42).

### Sensitivity analysis

This study included the initial daily dose to reflect the baseline status of participants, while the average daily dose of the whole maintenance period was also a determinant for relapse. Therefore, we alternatively included the average daily dose of the whole maintenance period in the group lasso regression models for both treatment durations to examine its significance on relapse.

All the analyses were performed using R 4.1.0 (R Foundation for Statistical Computing, Vienna, Austria). The BN models were visualized by Netica 5.18 (Norsys Software Corp., Vancouver, BC, Canada).

## Results

**Figure 1:**
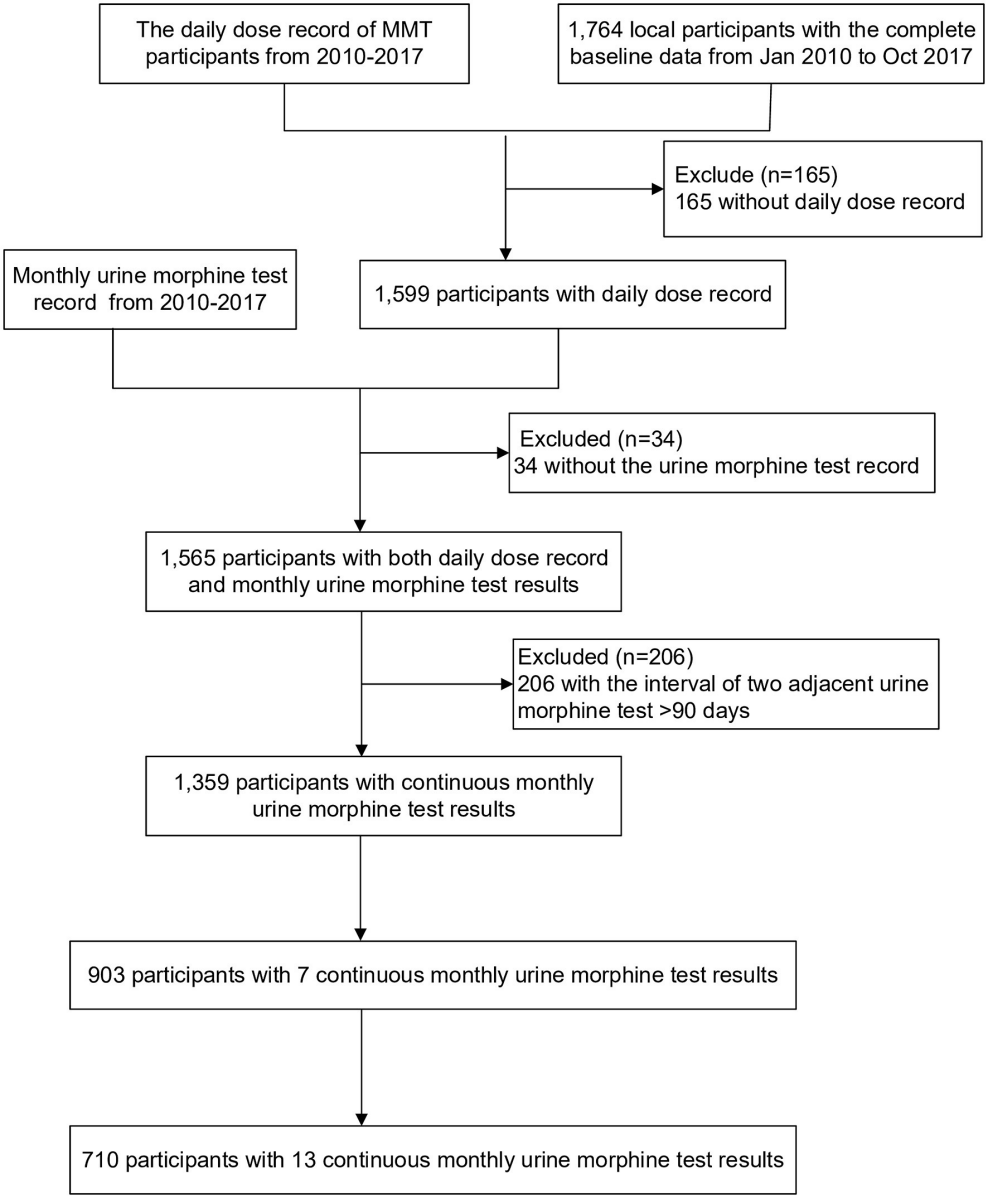
Flow chart of participants' inclusion. Excluded because of the incomplete data, test results, or record of daily methadone dose.

From January 2010 to April 2017, a total of 903 newly enrolled participants with completed records received MMT for over 6 consecutive months, 710 of whom have received treatment for 12 months. The detailed process of participant inclusion is displayed in Figure 1. We found that 32.0% (289) of them relapsed during the first 6 months. Among those who persisted in MMT for 12 months, 39.0% of them (277) relapsed in the process. This was significantly more than those who received 6-month treatment (*P* = 0.003, Table 1) and only 27.8% of the relapses occurred in the last 6 months. Supplementary Tables 1, 2 showed the baseline characteristics of participants of both treatment durations.

**Table 1:** The proportion of relapse among newly enrolled participants who received 6 and 12 months of consecutive MMT in seven clinics in Guangdong Province.

	**No. (%)**				
**Treatment**	**Relapse**		**Total**	χ^2^	***P***
**duration**	**Yes**	**No**			
6 months	289 (32.0)	614 (68.0)	903	8.58	0.003
12 months	277 (39.0)	433 (61.0)	710		

### The variables selection through the group lasso regression

**Figure 2:**
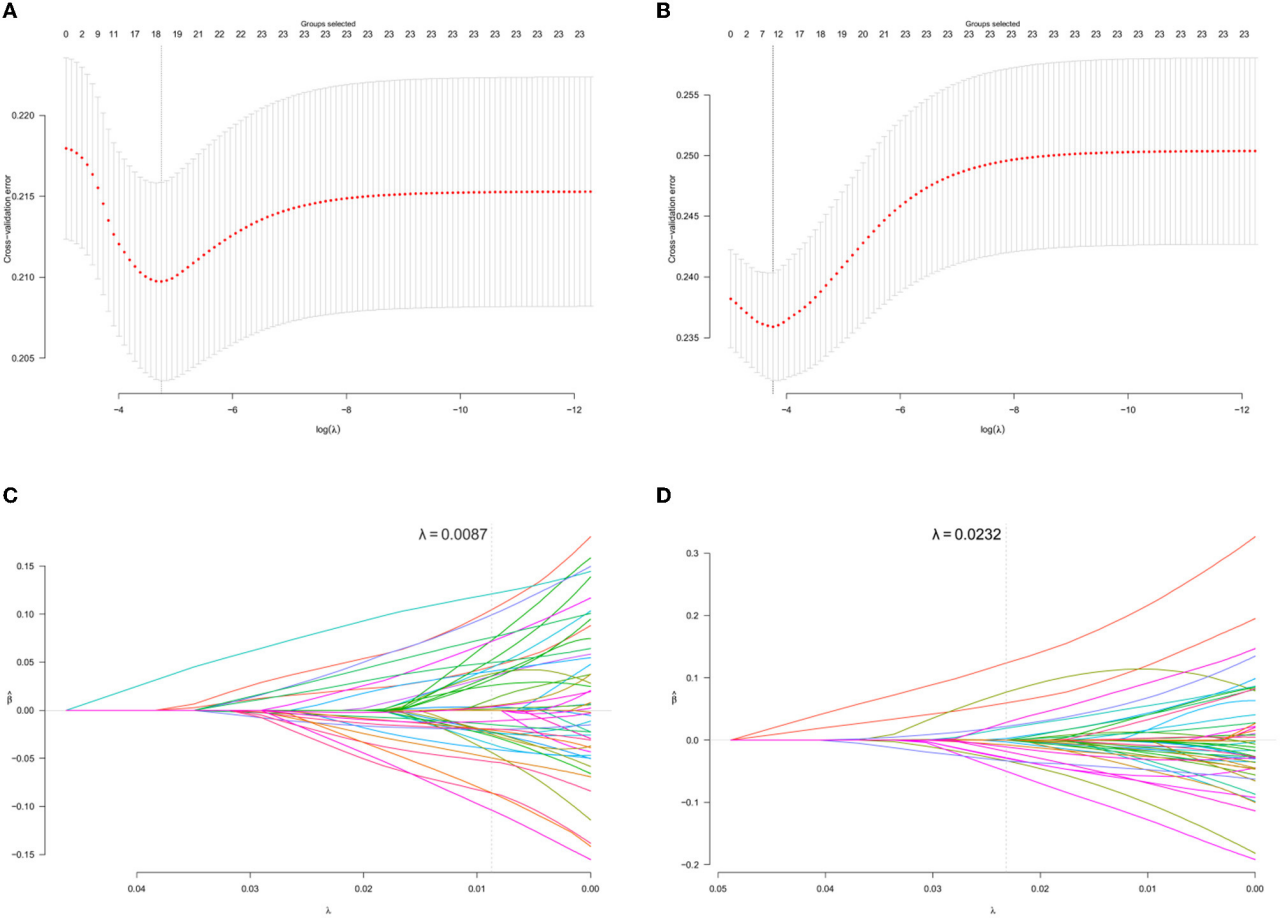
Variables selection using the group lasso regression. **(A, B)** present the 10-fold cross-validation error of the parameter λ based on the baseline data of newly enrolled MMT participants who received the 6- and 12-month consecutive treatments, respectively. **(C, D)** present the parameter solution path based on the baseline data of newly enrolled MMT participants who received the 6- and 12-month consecutive treatments, respectively. The gray dashed line denotes the optimal λ of each model. Every colored line means a single variable and those that the gray passed across were selected.

The optimal parameter λ was specified in the group lasso regression through the 10-fold cross-validation error and was used to select significant variables (Figure 2). A total of 18 groups of variables were considered as risk factors for participants who have received the 6-month treatment, while eight groups of variables were chosen for those who have received the 12-month treatment. The coefficients of variables are presented in Supplementary Table 3, where 0 indicates the statistical insignificance.

### BN model for the risk assessment of relapse

**Figure 3:**
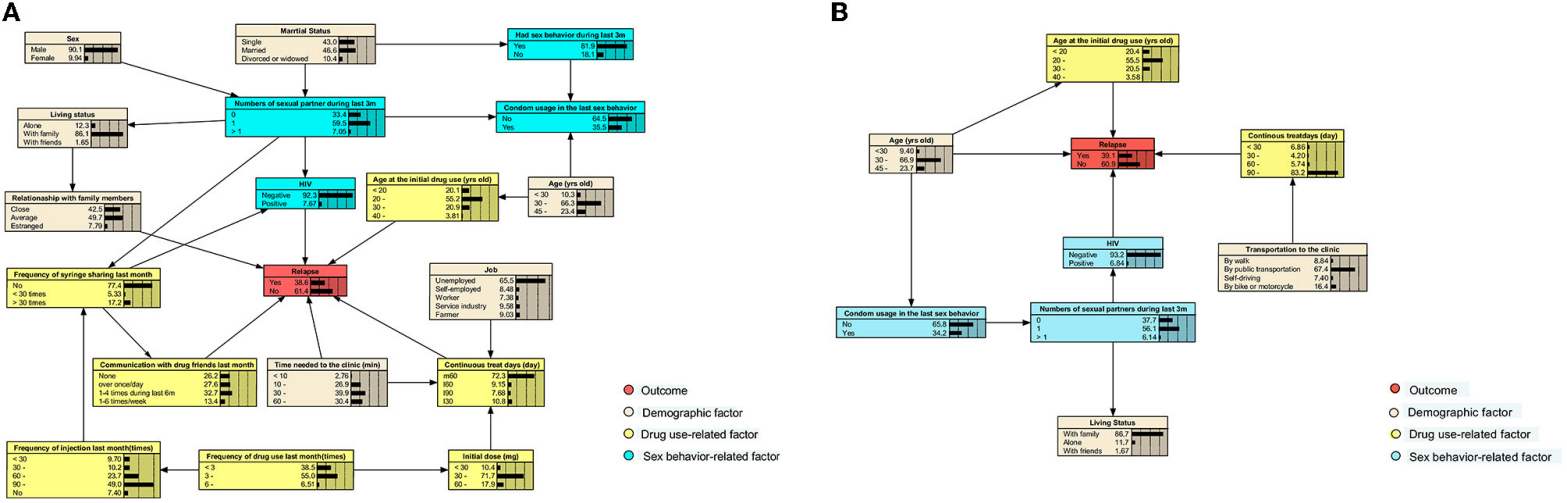
Adjusted BN model of factors related to relapse among participants receiving the 6- and 12-month consecutive MMT. Each color indicates a particular type of variable. **(A)** The adjusted BN model of factors for relapse of MMT participants after the first 6-month treatment. **(B)** The adjusted BN of that during the first 12-month treatment.

The initial BN models for 6- and 12-month treatments were constructed using the Tabu-search algorithm (Supplementary Figures 2, 3) and were then adjusted based on the published literature and experience of experts in this field (Figure 3). The nodes were clarified into four types, which comprised outcome (relapse), demographic factors, drug use, and sex behavior-related factors.

**Figure 4:**
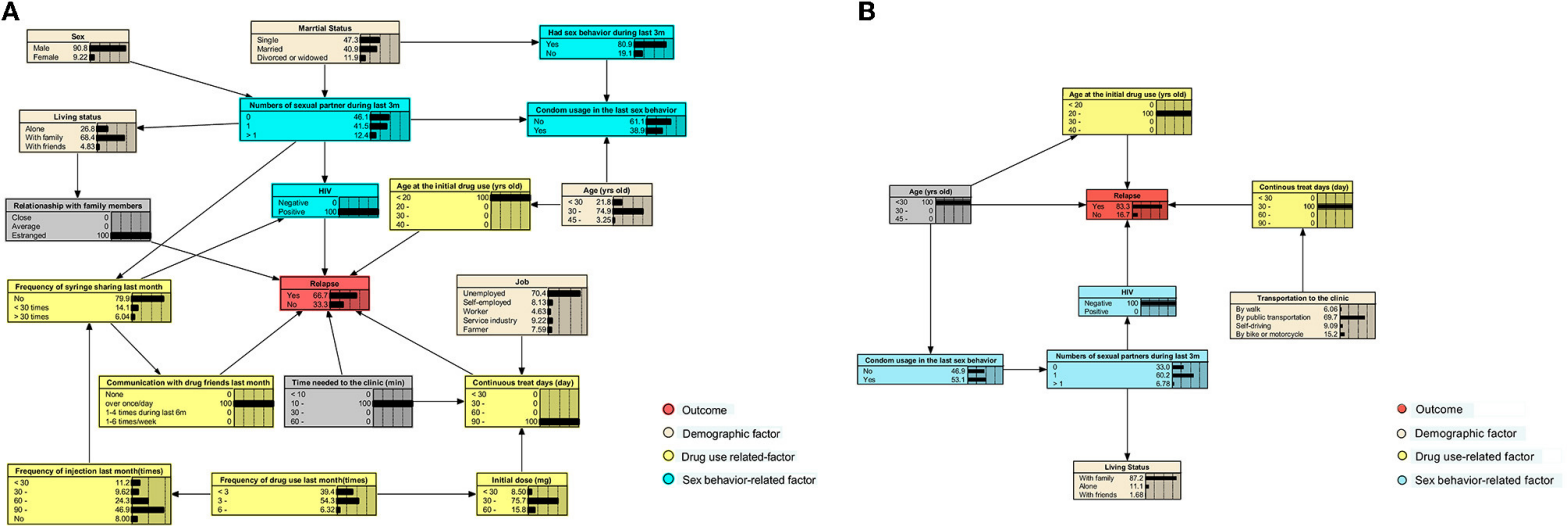
BNs show the highest risk of relapse for participants who have received 6- and 12-month consecutive MMT. **(A)** The BNs that lead to the highest risk of relapse in participants during the first 6 months of treatment. **(B)** The BNs that lead to the highest risk during the first 12 months of treatment.

For participants who have received the 6-month MMT, we found that HIV infection status, age at the initial drug use, continuous treatment days, the time required to reach the clinic, communication with drug friends in the last month, and the relationship with family members were directly related to relapse. Those who were HIV-positive, first used drugs before 20 years of age, had an estranged relationship with family, communicate with friends who used drugs at least once a week, and had received treatment for more than 90 days consecutively had the highest possibility (66.7%) of relapse (Figure 4A).

When the treatment duration was extended to 12 months, only age, age at the initial drug use, HIV infection status, and continuous treatment days were directly associated with relapse. In this case, HIV-negative participants, who were below 30 years of age, first took drugs at the age of 20–30 years, and received MMT for 30–60 days since the last drop-out, would have the highest relapse risk of 83.3% (Figure 4B). When a participant who received the 6-month treatment was under the same situation mentioned earlier, the risk would decrease to 46.3% (Supplementary Figure 4).

We also investigated the conditional probabilities of nodes that only were significant in either the 6- or 12-month model, including jobs, initial daily doses, the time needed to the clinic, and the transportation they took to the clinic. Specifically, among those who were treated for 6 consecutive months, farmers (40.1%), participants who took < 30 mg per day at the initial stage (40.2%), and who needed < 10 min to reach the clinic (48.3%) were predicted to have the highest risk of relapse (Table 2). The above groups also accounted for the largest proportion among those with the shortest retention (<30 continuous treatment days), compared with other subgroups, which were 17.0, 14.3, and 19.3%, respectively (Supplementary Table 6).

**Table 2:** Conditional probability distribution of relapse with the job, initial daily dose, and time needed to the clinic.

**Node**		**Relapse (%)**	
		**Yes**	**No**
Initial daily dose (mg)	<30	40.2	59.8
	30–60	38.2	61.8
	>60	39.1	60.9
Job	Unemployed	38.0	62.0
	Self-employed	39.6	60.4
	Worker	39.8	60.2
	Service industry employee	38.9	61.1
	Farmer	40.1	59.9
Time needed to the clinic (min)	<10	48.3	51.7
	10–	40.8	59.2
	30–	37.7	62.3
	60–	36.9	63.1

^*^The probability was obtained based on the BN model of 6-month treatment duration.

As for participants who received MMT for 12 months, the transportation that they took to the clinic was an additional factor that impacted relapse. Those who drove to the clinic were most possible to relapse (39.6%, Table 3) and to have retention of fewer than 30 days (10.7%, Supplementary Table 6), while those who walked to the clinic had the lowest risk.

**Table 3:** The conditional probability distribution of relapse with transportation and time needed to the clinic as parent nodes.

**Parent node**		**Relapse (%)**	
		**Yes**	**No**
Transportation	By walk	38.7	61.3
	By public transportation	39.0	61.0
	By bike or motorcycle	39.1	60.9
	Self-driving	39.6	60.4

^*^The probability was obtained based on the BN model of 12-month treatment duration.

### Evaluation of model performance

**Figure 5:**
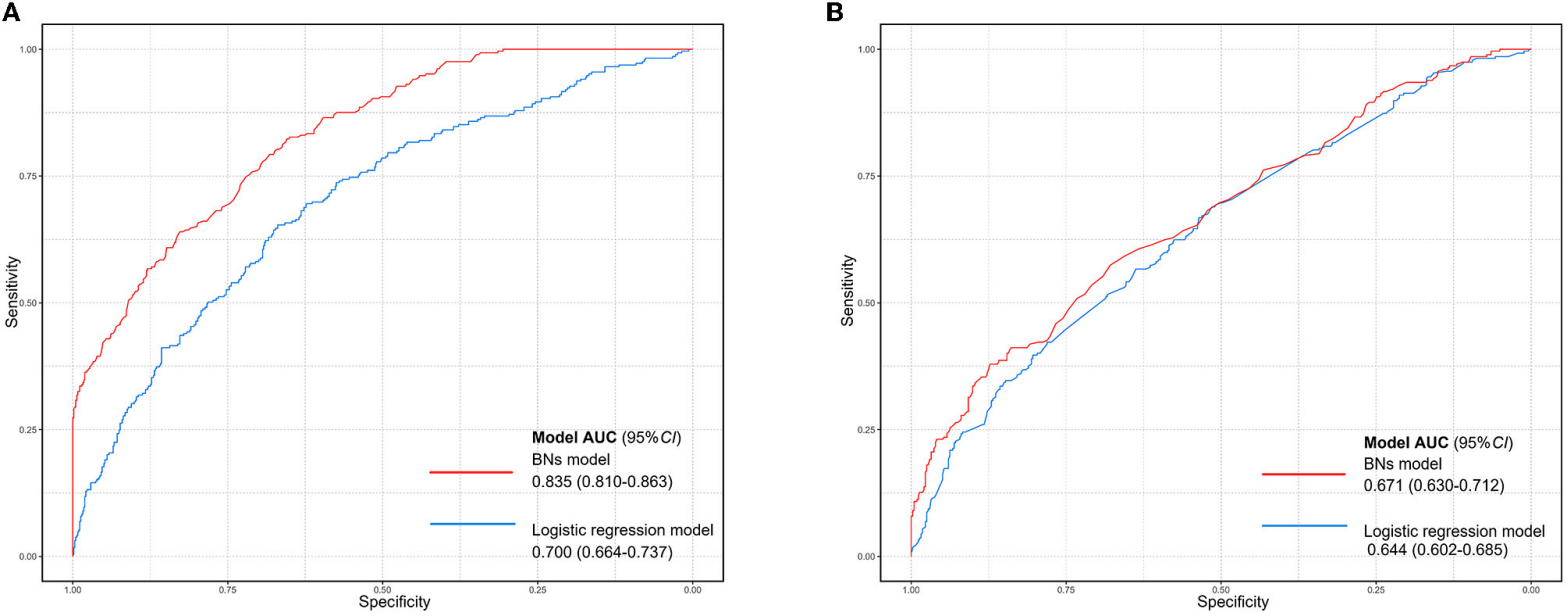
Comparison of ROC curves between the group lasso logistic model and the BN model. **(A)** The ROC curve of two models based on the baseline data of newly enrolled participants receiving MMT for 6 months. **(B)** The ROC curve of two models based on the baseline data of newly enrolled participants receiving MMT for 12 months. The blue curve indicates the group lasso logistic model and the red curve indicates the BN model.

The group lasso is always combined with logistic regression when the dependent variable is binary. Thus, we compared the group lasso logistic model and the group lasso BNs model, using the AUC value. Figure 5 illustrated that the BNs model had better performance (6-month model: AUC = 0.835, 95%CI: 0.810–8.863; 12-month model: AUC = 0.659, 95%CI: 0.630–0.712) than the logistic model (6-month model: AUC = 0.700, 95%CI: 0.664–0.737; 12-month model: AUC = 0.644, 95%CI: 0.602–0.685) in this study. The parameter estimation result of the ROC curve is presented in Supplementary Table 7.

### The variable selection results of sensitivity analysis

For the model of those who received the 6-month MMT, the initial daily dose was considered significant in the main result while the average daily dose was not in the sensitivity analysis. Apart from these, the other selected variables were the same as that of the main text, with little difference in the coefficients of variables between the two analyses. For the 12-month treatment, the average daily dose was not considered significant, being the same as that of the initial dose in the main result. The cross-validation results are presented in Supplementary Figure 1, and the coefficients of variables are presented in Supplementary Table 4.

To sum up, most results of sensitivity analysis were similar to those of the main results, which confirmed the robustness of our findings. The average daily dose was considered insignificant in the group lasso models of both treatment durations, while the initial daily dose was significant in the model of 6-month treatment duration. This supported our finding that the initial dose is important for participants at an early stage.

## Discussion

This study found that the relapse risk of newly enrolled MMT participants might increase with the treatment duration and risk factors differed from those who have received the 6- and 12-month consecutive treatments.

It is believed that the maximum effectiveness of MMT starts to show up at least after 12 months (2); however, participants tend to relapse at the early stage because of withdrawal symptoms, side effects, and a strong craving for drugs (43). Most relapses happened in the first 6 months (12) and this was confirmed by our study. Nonetheless, the relapse risk might rise when the participants were treated for a longer time. According to our findings, participants who received the 12-month treatment had a higher possibility of relapse (39%), compared with those who received the 6-month treatment (32%), and this was consistent with other research (16).

Relapse is a multifactorial outcome (44), which has been proven to be correlated with factors, such as marital status, living status, relationship with family, and drug use behavior (12, 16, 45, 46). This study implied that these factors were influential for participants who completed the 6-month consecutive MMT. Specifically, living alone or being estranged from family and friends might indicate that the participants received little social support, which made them more likely to drop out and relapse (47, 48). This could be more apparent among Chinese participants because of their family-oriented culture (49). They may be impacted by poor family relationships more greatly, causing mental health or economic issues. Both of these will increase the risk of relapse. Previous drug-use habits are associated with relapse as well (47). For instance, people who used drugs more frequently before MMT and exhibit high-risk behaviors such as syringe sharing might feel difficult to adapt to the substitution of methadone. Severe withdrawal symptoms could also lead them to return to heroin use (50). In addition, the daily methadone dose also counts for relapse, especially at the initial stage of MMT (12, 51). Komasi et al. (12) found that participants who relapsed during the first 6-month treatment reported more dose non-satisfaction than those who did not. A low methadone dose is a leading risk factor for relapse and could hinder the effectiveness of MMT. In this study, participants who took <30 mg of methadone per day during the first 6 months had the lowest retention and the highest relapse risk. Therefore, we suggest conducting interventions about family engagement and in-time dose adjustment for those who are at the early stage of MMT.

However, the effects of these factors will be fading with the treatment duration in line with our findings. This could be explained by several reasons: first, pleasant family relationships and reduced communication with drug friends could engage better compliance of participants, reflected by longer treatment duration. Second, the craving for drugs would decrease with treatment. Therefore, previous drug-use behaviors might gradually become less of a determinant for those with longer treatment duration. Finally, those who were treated for 12 months might have been taking a more appropriate dose at the beginning of the treatment than those with short retention, which decreased the possibility of suffering the withdrawal symptoms and helped with a quick adaptation to methadone.

In contrast, age, the age at the initial drug use, the HIV infection status, sexual behavior, and continuous treatment days had long-lasting effects on relapse, regardless of the treatment duration. Among these factors, age and age at the initial drug use could reflect the severity of the drug addiction history. A long addiction history is usually related to a high risk of relapse, and this has been reported among participants at different stages of treatment (49, 52). Adjusting to MMT might be harder for those who were addicted to heroin at a younger age (53). In addition, having unprotected sex and multiple sexual partners increases the relapse risk as they are strongly associated with HIV infection, and HIV-positive participants are believed to have poorer compliance with MMT (54). Some antiretroviral medications can reduce the potency of methadone (55), which makes it harder for them to adapt to MMT. Therefore, high-risk sexual behaviors are related to a greater probability of relapse. In addition, short continuous treatment days indicate poor adherence because of which relapse is always a dominant cause (18, 56). Our findings are consistent with the aforementioned conclusions, and we, therefore, suggest implementing health education for preventing HIV/STI and anti-narcotics in the whole process of treatment to promote adherence and reduce the relapse rate.

We also identified the groups with the highest relapse risk for participants of both durations. Among those who persisted with 6-month MMT, if they were HIV-positive, addicted to drugs since their teenage, communicated with friends more than once a day, and had poor family supports, they would have the highest possibility of relapse (66.7%). In contrast, the riskiest group among those who received the 12-month MMT was HIV-negative participants who were younger than 30 years and started to use drugs after 20 years of age, with continuous treatment of 30–60 days. They were predicted to have a relapse risk as high as 83.3%. Being inconsistent with other research, we did not find being HIV-positive was a risk for relapse in this group. It might be because the number of relapsed and non-relapsed HIV-positive participants was the same (23 vs. 23, see Supplementary Table 2), and no statistical significance was detected between the groups. Apart from this, other findings that young participants who were addicted to drugs at an early age with poor compliance to MMT were at a risk of relapse, which was confirmed by previous research (56, 57).

If participants who have received the 6-month MMT were under the same condition leading to the highest relapse risk for those who received the 12-month MMT, the relapse risk would be 46.3%, nearly half of that of the latter. This might be because the occurrence of relapse during the first 6-month treatment was associated with more factors, compared with those with longer treatment; thus, the effects of common factors might be weakened.

There are also some highlights in this study. For example, we found that those who drove or needed <10 min to reach the clinic were more likely to relapse than other participants. Theoretically, the long distance and traveling difficulties to the clinic would reduce adherence to MMT (58, 59). However, the high accessibility such as living too close to the clinic means having the flexibility in choosing the time to get there, which might decrease adherence as well. In addition, being capable of driving indicates that participants might have a relatively high income or well-being life. They have a greater possibility to buy drugs such as heroin, compared to those who were less paid or unemployed.

Another unexpected finding is that it was farmers, rather than the unemployed, who were most vulnerable to relapse. A rational interpretation could be that, unlike those who live in towns, people who live in rural areas might have poorer accessibility to MMT services. Research conducted in Thailand showed that rural residents faced barriers to utilizing MMT services, including missing the opening hours of clinics and the unaffordable cost of travel (60). Harm Reduction International (1) also highlighted that rural communities were underserved by harm reduction services and this geographical gap has hindered the implementation of MMT among rural residents, including farmers. Hence, more adaptive operations are required to improve the accessibility of MMT for this group.

Based on the findings mentioned earlier, we, herein, provided several recommendations for future policy-making:

1) The influence of treatment durations should be considered when implementing or evaluating the effectiveness of relevant interventions or strategies.
2) The future guideline for methadone dosage adjustment should specifically consider the participants at the early stage of MMT.
3) The continuity of health education on HIV/STI and anti-narcotics should be emphasized when implementing related interventions.

In addition, we also listed several clinical implications based on the findings, which hopefully could be referenced for healthcare providers:

1) Family engagement and in-time dosage adjustment should be emphasized for those who received short-term MMT.
2) Health education on HIV/STI and anti-narcotics should be insisted on in the whole process of MMT.
3) High-risk groups, such as farmers and those who can easily assess MMT require more attention to prevent relapse.

To our knowledge, this study is the first one that revealed the difference in risk factors of relapse between participants who have received the short-term (6-month) and long-term (12-month) MMT. This enriched the previous findings, which only treated all the participants as a whole, regardless of the treatment duration. In addition, this study first applied the group lasso-based Bayesian network to assess the relapse risk of MMT participants. These methods have been popular in health research and excel in dealing with the dependence on variables, which is the issue to be solved in this study. Performing these methods filled the gap in methodology in the field of MMT.

Several limitations exist in this study. First, this was a retrospective study that used secondary data. This type of study design will have issues, such as the absence of confounders, selection bias, and less timeliness (61–63). Particularly, we did not include factors such as mental health status (64) or brain function (65), which were also considered to be influential to relapse. The latest data can only be dated back to 2017. These may impact the replicability and robustness of our findings. However, the questionnaire from which we obtained the data was designed by the national MMT working group and filled by the professional clinic staff for quality control. It also covered the majority of aspects of the participants' information and most MMT-related research in China was conducted using data from this questionnaire. Therefore, its rigor and authority can be ensured. Second, the sample is not that representative as all the participants were from seven MMT clinics in Guangdong Province. It needs to be cautious when generalizing our findings to other contexts. The sample size is not large enough either and this made the BN model of 12 months less discriminative, evidenced by the lower AUC. All these issues could be improved once we obtained more data from more settings.

## Conclusion

In summary, participants receiving MMT for a long duration may be generally at a higher risk of relapse than those of short duration. Factors including personal living status, previous drug-use behaviors, and daily methadone dose would become less significant as the treatment continued. However, the duration of drug-use history, sexual behaviors, HIV infection status, and adherence to MMT would remain influential in the long term. Therefore, we recommend implementing interventions about family engagement and in-time dose adjustment for those who attended MMT for a short term and conducting health education for preventing HIV/STI and anti-narcotics in the whole process. More focus should be paid to farmers and those who have high accessibility to MMT services. In this manner, participants could receive more targeted treatment, contributing to reducing the relapse rate and improving compliance with MMT.

## Data availability statement

The data analyzed in this study is subject to the following licenses/restrictions: The data supporting the findings of this study are not publicly available to protect the confidentiality and privacy of participants. Requests to access these datasets should be directed to lingli@mail.sysu.edu.cn.

## Ethics statement

The study was reviewed and approved by the Institutional Review Board of the School of Public Health, Sun Yat-sen University, Guangzhou, China (No. 2020-39).

## Author contributions

XT and LL conceptualized and designed the study. XT, CF, and CX completed the data cleaning and formal analysis. XT wrote the original draft. XT, CF, WW, CW, and ZC contributed to reviewing and editing the manuscript. LL provided the funding for this study. All the authors have read and approved the final manuscript.
