# Supplementary material for: The risk assessment of relapse among newly enrolled participants in methadone maintenance treatment: A group-LASSO based Bayesian network study
Source: Front Public Health. 2023 Jan 17;10:1032217. doi: 10.3389/fpubh.2022.1032217 (PMC9886899; doi:10.3389/fpubh.2022.1032217)
Supplement: Supplementary file 1 [file Data_Sheet_1.pdf]

Supplementary Material

Table S1. The baseline demographic, drug usage and sex behavior characteristics of newly-enrolled MMT participants under the treatment for 6 consecutive months

Table S2. The baseline demographic, drug usage and sex behavior characteristics of newly-enrolled MMT participants under the treatment for 12 consecutive months

Table S3. Coefficients of selected variables using group-lasso regression under different treatment durations

Figure S1. Variables selection using Group-lasso regression for sensitivity analysis.

Table S4. Coefficients of selected variables using group-lasso regression under differenttreatment durations (replaced“initial daily dose”with“average daily dose”)

Figure S2. The initial model of Bayesian network of factors relating to relapse of newly enrolled MMT participants who have received 6-month treatment

Figure S3. The initial model of Bayesian network of factors relating to relapse of newlyenrolled MMT participants who have received 12-month treatment

Figure S4. The Bayesian network of participants who have received 6-month consecutive MMT under the conditions leading to the highest relapse risk of that of 12-month consecutive MMT

Table S5. Conditional probability distribution of continuous treatment days with job, initial daily dose and the time needed to the clinic as parent nodes

Table S6. The conditional probability distribution of continuous treatment days with transportation as parent nodes

Table S7. The model comparison of group-LASSO logistic and Bayesian networks basedon different treatment durations

Table S1. The baseline demographic, drug usage and sex behavior characteristics of newly-enrolled MMT participants under the treatment for 6 consecutive months

Variable	No. (%)			χ^2	*P*
	Total	Relapse			
		No (%)	Yes (%)		
		614 (67.9)	289 (32.1)	-	-
**Demographic Characteristics**					
**Age** (years old)					
< 30	92 (10.2)	71(11.6)	21(7.3)	10.83	0.004*
30 - 44	600(66.4)	417(67.9)	183(63.3)		
>45	211(23.4)	126(20.5)	85(29.4)		
**Sex**					
Male	814(90.1)	559(91.0)	255 (88.2)	1.74	0.187
Female	89 (9.9)	55(9.0)	34(11.8)		
**Marital Status**					
Single	389(43.1)	256(41.7)	133(46.0)	4.27	0.118
Married	421(46.6)	300(48.9)	121(41.9)		
Divorced or widowed	93(10.3)	58(9.5)	35(12.1)		
**Job**					
Unemployed	594(65.8)	398(64.8)	196(67.8)	5.95	0.203
Service industry employee	86(9.5)	52(8.5)	34(11.8)		
Worker	66(7.3)	50(8.1)	16(5.5)		
Farmer	81(9.0)	58(9.4)	23(8.0)		
Self-employed and others	76(8.4)	56(9.1)	20(6.9)		
**Living status**					
Living alone	109(12.1)	64(10.5)	45(15.6)	6.99[a]	0.030*
With family	782(86.6)	544(88.6)	238(82.4)		
With friends	12(1.3)	6(1.0)	6(2.1)		
**Source of living expenses**					
Salary	403(44.6)	280(45.8)	122(42.2)	2.13	0.345
Family members	477(52.8)	309(52.1)	157(54.3)		
Social welfare and others	23(2.6)	13(2.1)	10(3.5)		
**Relationship with family**					

Close	385(42.6)	269(43.8)	116(40.1)	2.52	0.284
Average	450(49.8)	304(49.5)	146(50.5)		
Estranged	68(7.5)	41(6.7)	27(9.3)		
**Currently employed**					
No	562(62.3)	382(62.2)	180(62.3)	0.00	0.984
Yes	341(37.7)	232(37.8)	109(37.7)		
**Transportation to the clinic**					
By walk	69(7.7)	42(6.9)	27(9.3)	1.87	0.599
By public transportation	599(66.3)	413(67.2)	186(64.4)		
By bike or motorcycle	151(16.8)	102(16.7)	49(17.0)		
Self-drive or others	84(9.3)	57(9.3)	27(9.3)		
**Cost on drugs last month (Yuan)**					
<100	125(13.9)	84(13.7)	40(14.2)	1.11	0.564
100 -	513(58.8)	343(55.9)	170(58.8)		
300 -	265(29.3)	187(30.4)	78(27.0)		
**Time needed to reach the clinic (min)**					
<10	24(2.7)	13(2.1)	11(3.8)	12.54	0.006*
10 -	243(26.9)	149(24.3)	94(32.5)		
30 -	361(40.0)	247(40.2)	114(39.4)		
60 -	275(30.5)	205(33.4)	70(24.2)		
**Urine test**					
Negative	36(4.0)	23(3.8)	13(4.5)	0.29	0.590
Positive	867(96.0)	591(96.2)	276(95.5)		
**HIV test**					
Negative	842(93.2)	583(95.0)	259(89.6)	8.87	0.003*
Positive	61(6.8)	31(5.1)	30(10.4)		
**HCV test**					
Negative	135(15.0)	94(15.4)	41(14.2)	0.19	0.659
Positive	768(85.0)	520(84.6)	248(85.8)		
**Drug use behavior**					
**Way of taking drugs**					
Snorting or Smoking	189(20.9)	128(20.8)	61(21.1)	0.65	0.722
Injecting	632(70.0)	427(69.6)	205(70.9)		
Both	82(9.1)	59(9.6)	23(8.0)		

**Frequency of daily drug use last month**					
<3 times	348(38.5)	234(38.1)	114(39.4)	1.78	0.412
3- times	497(55.0)	336(54.7)	161(55.7)		
6- times	58(6.4)	44(7.2)	14(4.8)		
**Injected drugs last month**					
No	188(20.8)	133(21.7)	55(19.0)	0.83	0.364
Yes	715(79.2)	481(78.3)	234(81.0)		
**Frequency of drug infection last month**					
None	65(7.2)	49(8.0)	16(5.5)	5.45	0.236
< 30	86(9.5)	51(8.3)	35(12.1)		
30 -	91(10.0)	64(10.3)	27(9.3)		
60 -	214(23.8)	141(23.0)	73(25.3)		
90 -	447(49.5)	309(50.3)	138(47.8)		
**Frequency of syringe sharing last month**					
None	720(79.7)	485(78.9)	235(81.3)	4.68	0.096*
<30 times	37(4.1)	21(3.4)	16(5.5)		
≥30 times	146(16.2)	108(17.6)	38(13.1)		
**Duration of drug use (years)**					
< 5	87(9.6)	65(10.6)	22(7.6)	7.55	0.056*
5 -	136(14.5)	89(14.5)	47(16.3)		
10 -	493(54.6)	345(56.4)	147(50.9)		
20 -	187(20.8)	114(18.6)	73(25.3)		
**Age of the initial drug use（years old）**					
<20	181(20.1)	122(19.9)	59(20.4)	3.52	0.318
20 -	502(55.6)	345(56.2)	157(54.3)		
30 -	188(20.8)	130(21.1)	58(20.1)		
40 -	32(3.6)	17(2.8)	15(5.2)		
**Initial daily dose (mg)**					
< 30	92(10.2)	58(9.4)	34(11.8)	5.73	0.057*
30 -	651(72.1)	435(70.8)	216(74.7)		
60 -	160(17.7)	121(19.7)	39(13.5)		
**Continuous treat days (day)**					
<30	78 (78.7)	44(7.2)	34(11.8)	10.34	0.016*

30 -	55 (6.1)	32(5.2)	23(8.0)		
60 -	43(4.8)	26(4.2)	17(5.9)		
90 -	727(80.5)	512(83.4)	215(74.4)		
**Communication with friends who are drug users last month**					
None	234(25.9)	168(27.4)	66(22.8)	15.80	0.001*
4-6 times /month	296(32.8)	219(35.7)	77(26.6)		
1-6 times/week	121(13.4)	77(12.5)	44(15.2)		
More than once/day	252(27.9)	150(24.4)	102(35.3)		
**Sexual behavior**					
**Had sex behavior ever**					
No	162(17.9)	120(19.6)	42(14.5)	3.35	0.067*
Yes	741(82.1)	494(80.4)	247(85.5)		
**Numbers of sexual partner during last 3 months**					
None	303(33.6)	196(31.9)	107(37.0)	10.47	0.005*
1	540(59.8)	386(62.9)	154(53.3)		
>1	60(6.6)	32(5.2)	28(9.7)		
**Used condom during the last sex behavior**					
No	584(64.7)	384(62.5)	200(69.2)	3.82	0.051*
Yes	319(35.3)	230(37.5)	89(30.8)		

Note: *p<0.1; [a]Fishers' exact test

Table S2. The baseline demographic, drug usage and sex behavior characteristics of newly-enrolled MMT participants under the treatment for 12 consecutive months

Variable	No. (%)				
	Total	Relapse			
		No	Yes	χ^2	*P*
		433(61.0)	277(39.0%)	-	-
**Demographic Characteristics**					
**Age** (years old)					
< 30	66(9.3)	49(11.3)	17(6.1)	14.23	0.001*
30 -	476(67.0)	300(69.3)	176(63.5)		
45 -	168(23.7)	84(19.4)	84(30.3)		
**Sex**					
Male	638 (89.9)	390(90.1)	248(89.6)	0.05	0.817
Female	72 (10.1)	43(9.9)	29(10.4)		
**Marital Status**					
Single	308(43.4)	179(41.3)	129(46.6)	4.69	0.096*
Married	327(46.1)	213(49.2)	114(141.2)		
Divorced or widowed	75(10.6)	41(9.5)	34(12.3)		
**Job**					
Unemployed	474(66.8)	280(64.7)	194(70.0)	4.99	0.288
Service industry employee	70(9.9)	40(9.2)	30(10.8)		
Worker	53(7.5)	36(8.3)	17(6.1)		
Farmer	55(7.7)	36(8.3)	19(6.9)		
Self-employed and others	58(8.2)	41(9.5)	17(6.1)		
**Living status**					
Living alone	81(11.4)	41(9.5)	40(14.4)	5.36[a]	0.069*
With family	620(83.8)	388(89.6)	232(83.8)		
With friends	9(1.3)	4(0.9)	5(1.8)		
**Source of living expenses**					
Salary	315(44.4)	196(45.3)	119(43.0)	3.01	0.222

Family members	378(53.2)	230(53.1)	148(53.4)		
Social welfare and others	17(2.4)	7(1.6)	10(3.6)		
**Relationship with family**					
Close	293(41.3)	173(40.0)	120(43.3)	3.35	0.187
Average	367(51.7)	234(54.0)	133(48.0)		
Estranged	50 (7.0)	26(6.0)	24(8.7)		
**Currently employed**					
No	447(63.0)	265(61.2)	182(65.7)	1.47	0.226
Yes	263(37.0)	168(38.8)	95(34.3)		
**Transportation to the clinic**					
By walk	60(8.5)	28(6.5)	32(11.6)	9.57	0.023*
By public transportation	485(68.3)	293(67.7)	192(69.3)		
By bike or motorcycle	115(16.2)	81(18.7)	34(12.3)		
Self-drive or others	50(7.0)	31(7.2)	19(6.9)		
**Cost on drugs last month (Yuan)**					
< 100	94(13.2)	53(12.2)	41(14.8)	3.20	0.202
100 -	403(56.8)	240(55.4)	163(58.8)		
300 -	213(30.0)	140(32.3)	71(26.4)		
**Time needed to reach the clinic (min)**					
< 10	17(2.4)	10(2.3)	7(2.6)	3.23	0.357
10 -	183(25.8)	102(23.6)	81(29.2)		
30 -	292(41.1)	181(41.8)	111(40.1)		
60 -	218(30.7)	140(32.3)	78(28.2)		
**Urine test**					
Negative	28(3.9)	17(3.9)	11(4.0)	0.00	0.976
Positive	682(96.1)	416(96.1)	266(96.0)		
**HIV test**					
Negative	664(93.5)	410(94.7)	254(91.7)	2.49	0.114
Positive	46(6.5)	23(5.3)	23(8.3)		
**HCV test**					
Negative	102(14.4)	59(13.6)	43(15.5)	0.49	0.482
Positive	608(85.6)	374(86.3)	234(84.5)		
**Drug use behavior**					

**Way of taking drugs**					
Snorting or Smoking	137(19.3)	81(18.7)	56(20.2)	0.35	0.840
Injecting	501(70.6)	309(71.4)	192(69.3)		
Both	72(10.1)	43(9.9)	29(10.5)		
**Frequency of daily drug use last month**					
<3 times	267(37.6)	162(37.4)	105(37.9)	0.15	0.929
3- times	391(55.1)	235(55.0)	153(55.2)		
6- times	52(7.3)	33(7.6)	19(6.9)		
**Injected drugs last month**					
No	141(19.9)	86(19.9)	55(19.9)	0.00	0.998
Yes	569(80.1)	347(80.1)	222(80.1)		
**Frequency of drug infection last month**					
None	55(7.7)	35(8.1)	20(7.2)	4.98	0.289
< 30	71(10.0)	35(8.1)	36(13.0)		
30 -	72(10.2)	46(10.6)	26(9.4)		
60 -	157(22.1)	100(23.1)	57(20.6)		
90 -	355(50.0)	217(50.1)	138(49.8)		
**Frequency of syringe sharing last month**					
None	718(79.7)	483(78.9)	235(81.3)	4.71	0.095*
<30 times	37(4.1)	21(3.4)	16(5.5)		
≥30 times	146(16.2)	108(17.6)	38(13.1)		
**Duration of drug use (years)**					
< 5	57(86.0)	38(8.8)	19(6.9)	13.19	0.004*
5 -	99(13.9)	64(14.8)	35(12.6)		
10 -	401(56.5)	257(59.4)	144(52.0)		
20 -	154(21.5)	74(17.1)	79(28.5)		
**Age of the initial drug use （Years old）**					
< 20	145(20.4)	90(20.81)	55(19.9)	11.33	0.010*
20 -	397(55.9)	23(55.29)	158(57.0)		
30 -	145(20.4)	97(22.4)	48(17.3)		

40 -	23(3.2)	7(1.65)	16(5.8)		
**Initial daily dose (mg)**					
<30	59(8.3)	37(8.5)	22(7.9)	0.56	0.755
30 -	517(71.8)	311(71.8)	206(74.4)		
60 -	134(18.9)	85(19.6)	49(17.7)		
**Average daily dose (dose)**					
<30	68(9.6)	49(11.3)	19(6.9)	4.21	0.122
30 -	377(53.1)	222(51.3)	155(56.0)		
60 -	265(37.3)	162(37.4)	103(37.2)		
**Continuous treat days (day)**					
<30	48(6.8)	26(6.0)	22(7.9)	6.26	0.100*
30 -	29(4.1)	12(2.8)	17(6.1)		
60 -	40(5.6)	24(5.5)	16(5.8)		
90 -	593(83.5)	371(85.7)	222(80.1)		
**Communication with drug friends last month**					
None	200(28.2)	122(30.4)	78(28.2)	5.93	0.115
4-6 times /month	227(32.0)	151(34.9)	76(27.4)		
1-6 times/week	97(13.7)	58(13.4)	39(14.1)		
More than once /day	186(26.2)	102(23.6)	84(30.3)		
**Sexual behavior**					
**Had sex behavior ever**					
No	128(18.0)	78(18.0)	50(18.1)	0.00	0.99
Yes	582(82.0)	355(82.0)	227(81.9)		
**Numbers of sexual partner during last 3months**					
None	268(37.7)	147(33.9)	121(43.7)	9.77	0.008*
1	400(56.3)	264(61.0)	136(49.1)		
＞1	42(5.9)	22(5.1)	20(7.2)		
**Used condom during the last sex behavior**					
No	468(65.9)	273(63.0)	195(70.4)	4.06	0.044*
Yes	242(34.1)	160(37.0)	82(29.6)		

Note: **p* <0.10; [a]Fishers' exact test

Table S3. Coefficients of selected variables using group-lasso regression under different treatment durations

Variable	Treatment duration	
	6 months	12 months
Sex: Female	0.036	0
Age (years old): 30 -	0.045	0.060
Age (years old):45 -	0.105	0.124
Marital Status: Married	-0.023	0
Marital Status: Divorced or widowed	0.003	0
Job: Serviced industry employee	0.045	0
Job: Workers	-0.024	0
Job: Farmer	-0.036	0
Job: Self-employed and others	-0.023	0
Living status: With friends	-0.040	-0.003
Living status: With family	0.041	0.003
Source of living expense: family	0	0
Source of living expense: social welfare and others	0	0
Relationship with family: Average	0.001	0
Relationship with family: Estranged	0.012	0
Age of the initial drug use (years old):20 -	-0.023	0
Age of the initial drug use (years old):30 -	-0.044	-0.033
Age of the initial drug use (years old) :40 -	0.038	0.077
Transportation to the clinic: by public transportation	0	-0.028
Transportation to the clinic: by bike or motorcycle	0	-0.050
Transportation to the clinic: by self-drive or others	0	-0.029
Urine test	0	0
HIV test	0.121	0.019
HCV test	0	0
Frequency of daily drug use last month:3 - times	0.005	0
Frequency of daily drug use last month:6 - times	-0.025	0
Cost on drugs last month: 100 -	0	0

Cost on drugs last month: 300 -	0	0
Frequency of syringe sharing last month: <30 times	0.026	0
Frequency of syringe sharing last month: ≥ 30 times	-0.027	0
Frequency of drug infection last month:< 30 times	0.073	0
Frequency of drug infection last month: 30 - times	0.032	0
Frequency of drug infection last month: 60 - times	0.053	0
Frequency of drug infection last month:< 90 times	0.036	0
Had sex behavior ever	0.072	0
Numbers of sexual partner during last 3 months: One	- 0.020	- 0.033
Numbers of sexual partner during last 3months: >1	0.099	0.022
Used condom during the last sex Behavior	- 0.050	- 0.008
Communication with drug friends last month: 4 - 6 times/ month	- 0.015	0
Communication with drug friends last month: 1 - 6 times/ week	0.050	0
Communication with drug friends last month: More than once/day	0.076	0
Time needed to reach the clinic (min):10 -	- 0.020	0
Time needed to reach the clinic (min):30 -	- 0.054	0
Time needed to reach the clinic (min):60 -	-0.086	0
Average dose of the first week(mg): 30 -	-0.021	0
Average dose of the first week(mg): 60 -	-0.086	0
Continuous treat days: 30 -	0.004	0.028
Continuous treat days: 60 -	-0.011	-0.011
Continuous treat days: 90 -	-0.104	-0.018

Figure S1. Variables selection using Group-lasso regression for sensitivity analysis. (a) and (b) presents the 10-fold cross-validation error of the parameter λ based on thebaseline data of newly enrolled MMT participants who received 6-month and 12-month consecutive treatment,respectively. (c) and (d) presents the parameter solution path based on the baseline data of newly enrolled MMT who received 6-month and 12-month consecutive treatment.

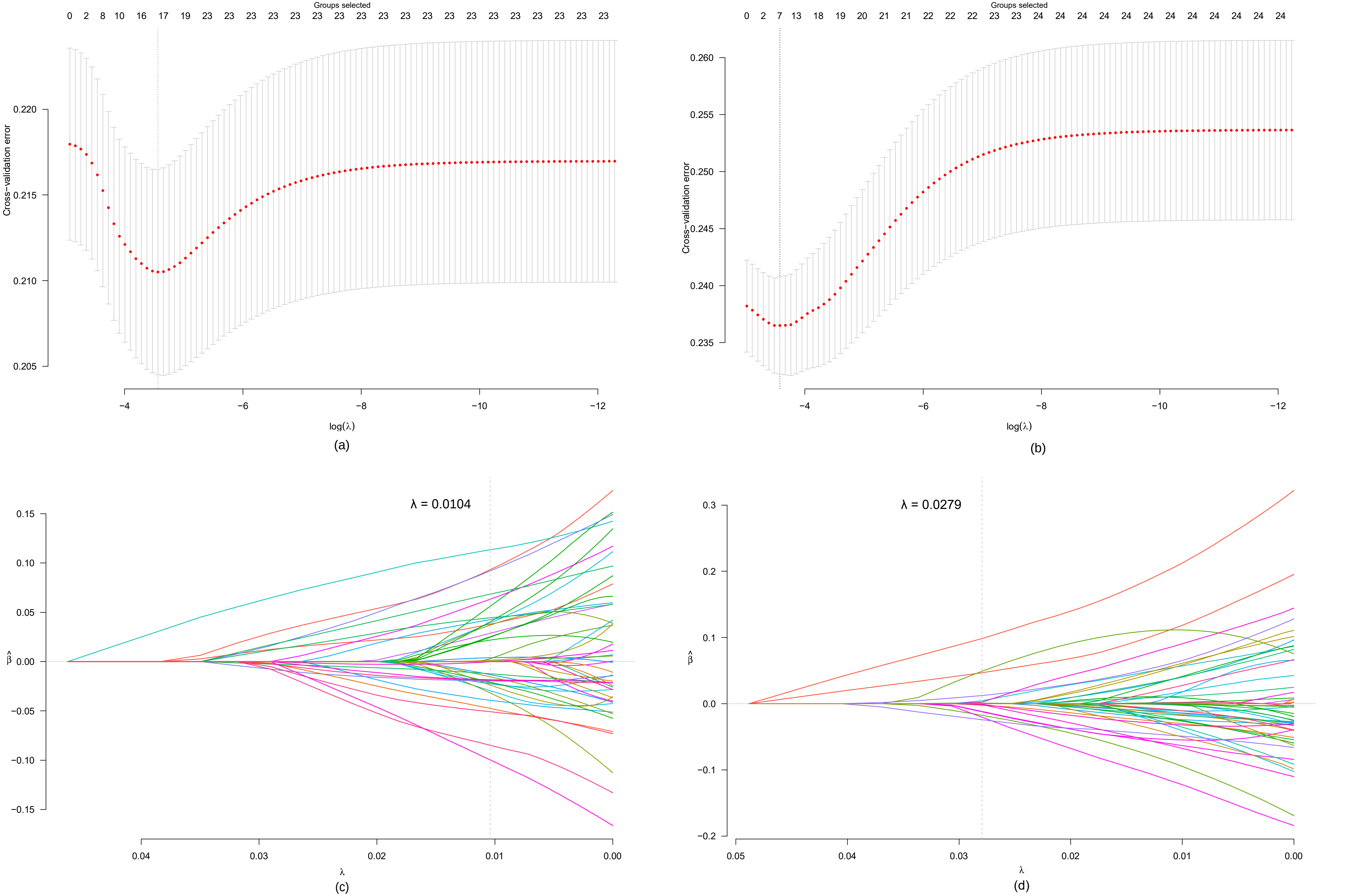

Table S4. Coefficients of selected variables using group-lasso regression under different treatment durations (replaced “initial daily dose” with “average daily dose”)

Variable	Treatment duration	
	**6 months**	**12 months**
Sex: Female	0.029	0
Age (years old): 30 -	0.038	0.047
Age (years old):45 -	0.093	0.098
Marital Status: Married	-0.013	0
Marital Status: Divorced or widowed	0.004	0
Job: Serviced industry employee	0.039	0
Job: Workers	-0.023	0
Job: Farmer	-0.029	0
Job: Self-employed and others	-0.022	0
Living status: With friends	-0.039	0
Living status: With family	0.042	0
Source of living expense: family	0	0
Source of living expense: social welfare and others	0	0
Relationship with family: Average	0.000	0
Relationship with family: Estranged	0.003	0
Age of the initial drug use (years old):20 -	-0.015	0.001
Age of the initial drug use (years old):30 -	-0.032	-0.017
Age of the initial drug use (years old) :40 -	0.037	0.049
Transportation to the clinic: by public	0	-0.012
Transportation to the clinic: by bike or motorcycle	0	-0.021
Transportation to the clinic: by self-drive or others	0	-0.012
Urine test	0	0
HIV test	0.113	0.004
HCV test	0	0
Frequency of daily drug use last month:3 - times	0.001	0
Frequency of daily drug use last month:3 - times	-0.024	0
Cost on drugs last month: 100 -	0	0
Cost on drugs last month: 300 -	0	0
Frequency of syringe sharing last month: <30 times	0.022	0
Frequency of syringe sharing last month: <30 times	-0.021	0
Frequency of drug infection last month: ≥30 times	0.056	0

Frequency of drug infection last month: 30 - times	0.024	0
Frequency of drug infection last month: 60 - times	0.040	0
Frequency of drug infection last month:< 90 times	0.025	0
Had sex behavior ever	0.063	0
Numbers of sexual partner during last 3 months: One	-0.020	-0.024
Numbers of sexual partner during last 3months: >1	0.092	0.012
Used condom during the last sex Behavior	-0.047	-0.002
Communication with drug friends last month: 4 - 6 times/ month	-0.012	0
Communication with drug friends last month: 1 - 6 times/ week	0.044	0
Communication with drug friends last month: More than once/day	0.068	0
Time needed to reach the clinic (min):10 -	-0.019	0
Time needed to reach the clinic (min):30 -	-0.051	0
Time needed to reach the clinic (min):60 -	-0.085	0
Average dose of MMT (mg): 30 -	0	0
Average dose of MMT (mg): 60 -	0	0
Continuous treat days: 30 -	0.000	0.002
Continuous treat days: 60 -	-0.018	-0.001
Continuous treat days: 90 -	-0.100	-0.001

Figure S2. The initial model of Bayesian network of factors relating to relapse of newly enrolled MMT participants who have received 6-month treatment

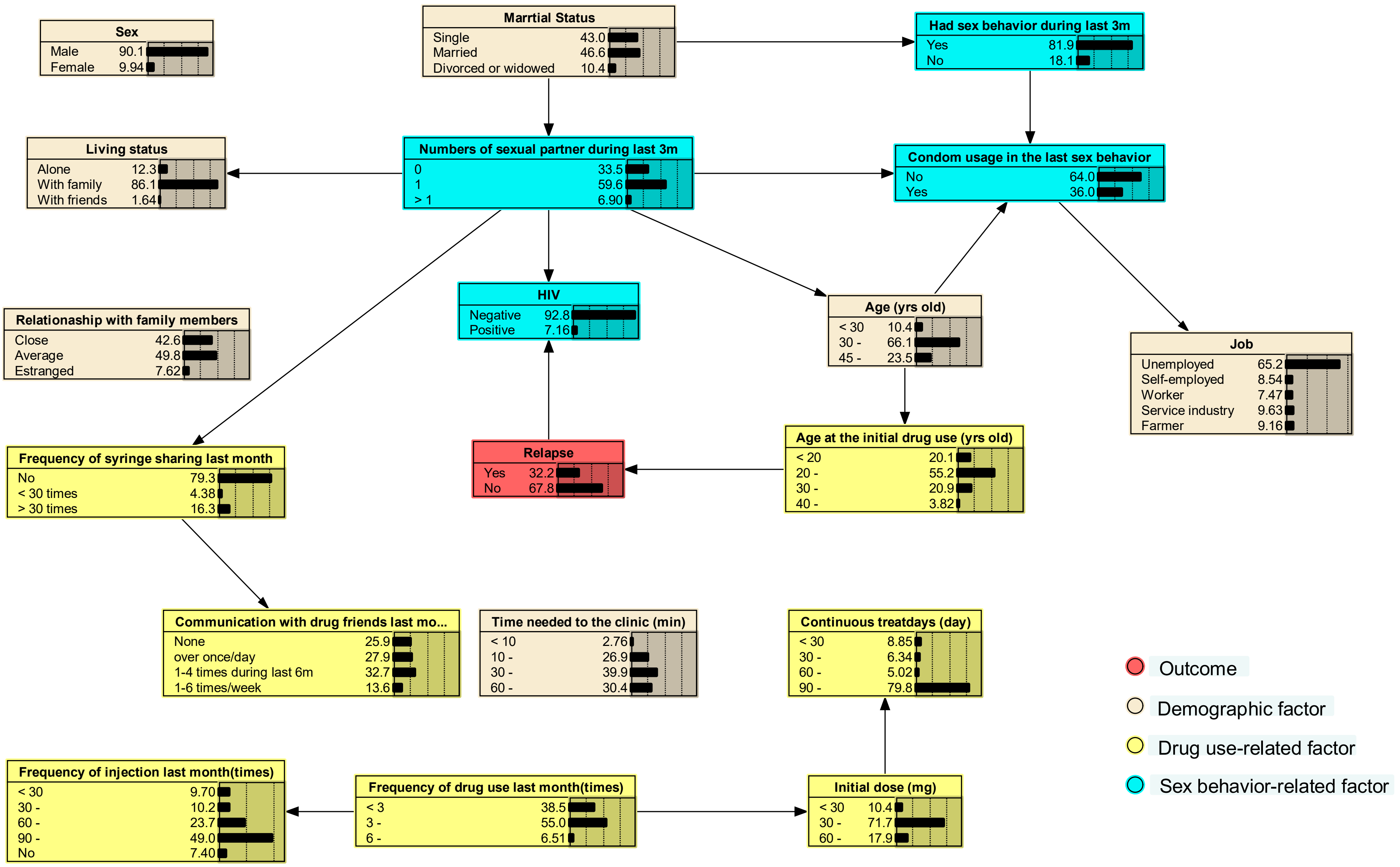

Figure S3. The initial model of Bayesian network of factors relating to relapse of newly enrolled MMT participants who have received 12-month treatment

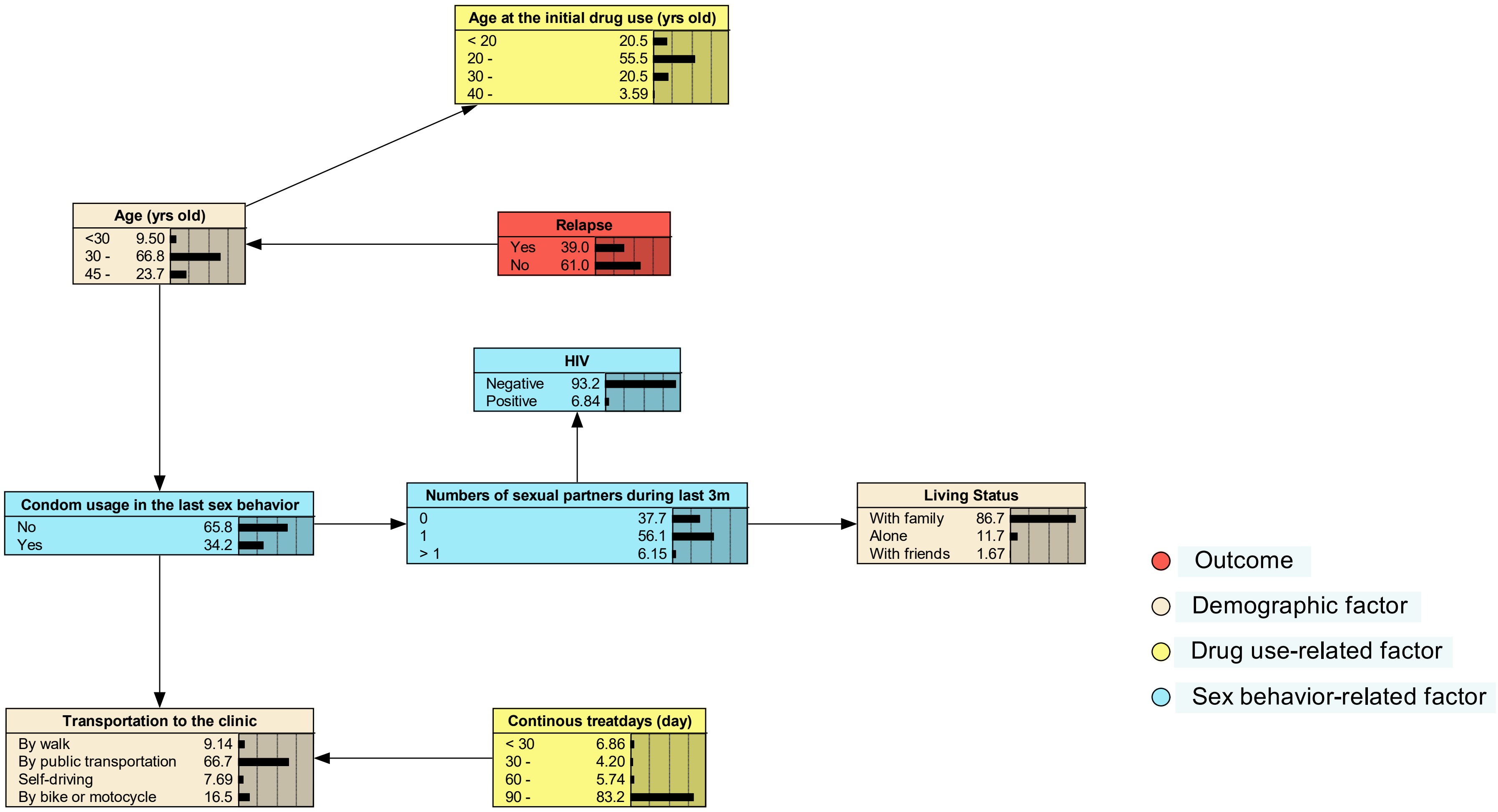

Figure S4. The Bayesian network of participants who have received 6-month consecutive MMT under the conditions leading to the highest relapse risk of that of 12-month consecutive MMT

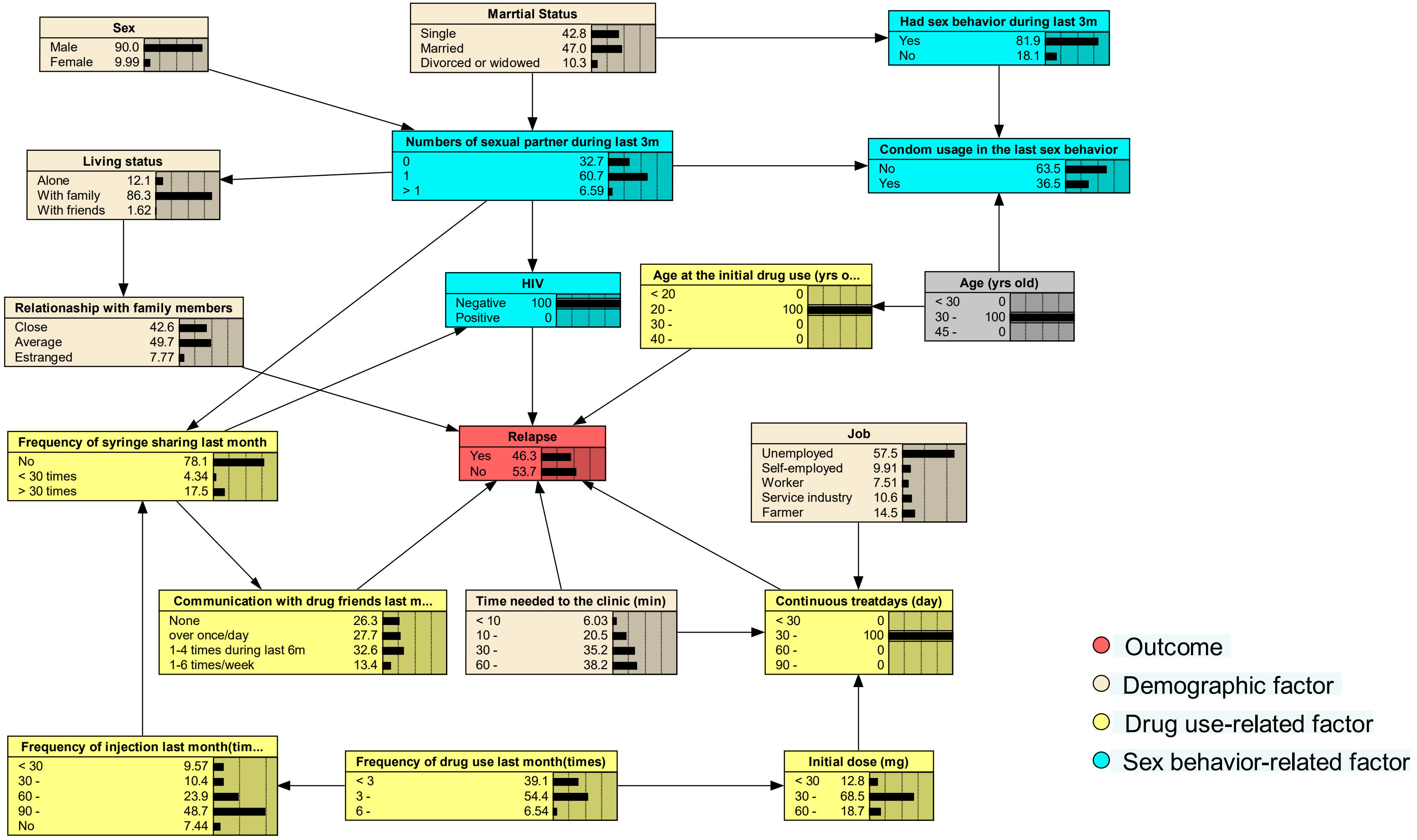

Table S5. Conditional probability distribution of continuous treatment days with job, initial daily dose and the time needed to the clinic as parent nodes

Parent node		Continuous treatment days (day, %)			
		< 30	30 - 60	60 – 90	> 90
Initial daily dose (mg)	< 30	17.0	11.3	12.7	59.0
	30 - 60	10.0	8.7	5.9	75.4
	> 60	10.7	9.6	11.7	68.0
Job	Unemployed	9.1	8.0	6.2	76. 7
	Self-employed	15.6	10.7	8.7	65.0
	Worker	18.5	9.3	10.4	61.7
	Service industry employee	9.2	10. 0	10.5	70.3
	Farmer	14.3	14.7	12.1	58.9
Time needed to the clinic (min)	< 10	19.3	20.0	15.0	45.7
	10 -	13.0	7.0	7.5	72.5
	30 -	9.6	8.1	7.6	74.7
	60 -	9.8	11.5	7.2	71.5

Note*： The probability was obtained based on the BN model of 6-month treatment

Table S6. The conditional probability distribution of continuous treatment days with transportation as parent nodes

Parent node		Continuous treatment days (day, %)			
		< 30	30 - 60	60 - 90	> 90
Transportation	By walk	6.0	2.9	4.4	86.7
	By public transportation	6.1	4.3	5.6	84.0
	By bike or motorcycle	8.8	3.9	5.6	81.7
	Self-driving	10.7	5.2	8.8	75.3

Note*： The probability was obtained based on the BN model of 12-month treatment

Table S7. The model comparison of group-LASSO logistic and Bayesian networks based on different treatment durations

**Model**	Group -Lasso logistic		Bayesian network	
**Treatment Duration**	6 months	12 months	6 months	12 months
**AUC (95%*CI*)**	0.700(0.664 -0.737)	0.644(0.602 -0.685)	0.835(0.810 -0.863)	0.671(0.630 -0.712)
**Sensitivity**	0.669	0.515	0.782	0.679
**Specificity**	0.654	0.690	0.696	0.574
